# Tissue culture-induced genetic and epigenetic alterations in rice pure-lines, F1 hybrids and polyploids

**DOI:** 10.1186/1471-2229-13-77

**Published:** 2013-05-05

**Authors:** Xiaoran Wang, Rui Wu, Xiuyun Lin, Yan Bai, Congdi Song, Xiaoming Yu, Chunming Xu, Na Zhao, Yuzhu Dong, Bao Liu

**Affiliations:** 1 Key Laboratory of Molecular Epigenetics of the Ministry of Education (MOE), Northeast Normal University, Changchun, 130024, China; 2 School of Bioengineering, Jilin College of Agricultural Science & Technology, Jilin, 132301, China; 3 Jilin Academy of Agricultural Sciences, Changchun, 130033, China; 4 Faculty of Agronomy, Jilin Agricultural University, Changchun, 13118, China; 5 School of Life Science, Changchun Normal University, Changchun, 130032, China; 6 Present address: Carnegie Institution for Science, Department of Plant Biology, Stanford University, Stanford, CA, 94305, USA

## Abstract

**Background:**

Genetic and epigenetic alterations can be invoked by plant tissue culture, which may result in heritable changes in phenotypes, a phenomenon collectively termed somaclonal variation. Although extensive studies have been conducted on the molecular nature and spectrum of tissue culture-induced genomic alterations, the issue of whether and to what extent distinct plant genotypes, e.g., pure-lines, hybrids and polyploids, may respond differentially to the tissue culture condition remains poorly understood.

**Results:**

We investigated tissue culture-induced genetic and epigenetic alterations in a set of rice genotypes including two pure-lines (different subspecies), a pair of reciprocal F1 hybrids parented by the two pure-lines, and a pair of reciprocal tetraploids resulted from the hybrids. Using two molecular markers, amplified fragment length polymorphism (AFLP) and methylation-sensitive amplified polymorphism (MSAP), both genetic and DNA methylation alterations were detected in calli and regenerants from all six genotypes, but genetic alteration is more prominent than epigenetic alteration. While significant genotypic difference was observed in frequencies of both types of alterations, only genetic alteration showed distinctive features among the three types of genomes, with one hybrid (N/9) being exceptionally labile. Surprisingly, difference in genetic alteration frequencies between the pair of reciprocal F1 hybrids is much greater than that between the two pure-line subspecies. Difference also exists in the pair of reciprocal tetraploids, but is to a less extent than that between the hybrids. The steady-state transcript abundance of genes involved in DNA repair and DNA methylation was significantly altered in both calli and regenerants, and some of which were correlated with the genetic and/or epigenetic alterations.

**Conclusions:**

Our results, based on molecular marker analysis of *ca.* 1,000 genomic loci, document that genetic alteration is the major cause of somaclonal variation in rice, which is concomitant with epigenetic alterations. Perturbed expression by tissue culture of a set of 41 genes encoding for enzymes involved in DNA repair and DNA methylation is associated with both genetic and epigenetic alterations. There exist fundamental differences among distinct genotypes, pure-lines, hybrids and tetraploids, in propensities of generating both genetic and epigenetic alterations under the tissue culture condition. Parent-of-origin has a conspicuous effect on the alteration frequencies.

## Background

Plant tissue culture, being comprised of sequential dedifferentiation (formation of callus) and re-differentiation (regeneration into plants) phases [1,2], represents a traumatic stress to plant cells and often provokes an array of genetic and epigenetic instabilities [3]. At least a portion of the genetic and/or epigenetic alterations can be manifested as heritable phenotypic changes, and which is collectively termed somaclonal variation [4]. Based on the complexity as well as the often genetic-context-dependent features of somaclonal variation, Phillips and colleagues (1994) proposed that somaclonal variation is a “self-imposed” mutagenesis, which can be largely attributable to the breakdown of normal cellular controls for genetic and epigenetic integrity [5].

Although extensive studies have been conducted on the molecular nature and spectrum of tissue culture-induced genomic alterations [6-11], whether and to what extent distinct plant genotypes, e.g., pure-lines, hybrids and polyploids, may respond differentially to the tissue culture condition remain to be fully understood.

The difference as well as its attendant biological effects between a hybrid (by extension an allopolyploid) genome and that of a pure-line are fundamental and myriad, as being reflected by both their distinct evolutionary trajectories as biological species and agricultural utilization as different crops. An issue bearing both theoretical and applied implications is whether and to what extent the distinct types of genomes, pure-line, hybrid and polyploid, are different under various environmental conditions [12]. Given the unique properties of plant tissue culture, mentioned above, it is of interest to compare the different types of genomes under the tissue culture condition with respect to genomic instability. Hitherto, this issue has been sparsely addressed.

We recently reported in sorghum (*Sorghum bicolor* L.) that there exists a sharp difference in the degree of both genetic and epigenetic instabilities at randomly sampled genomic loci under tissue culture between F1 hybrids and their parental pure lines, with the former being highly stable while the later highly mutable [13]. This trend, however, was not observed in a set of maize (*Zea mays* L.) inbred lines and their F1 hybrids, in which the frequencies of both genetic and epigenetic alterations were largely dependent on genotypes, and F1 hybrids were not more stable than inbred parents [14]. Although this discrepancy can be explained by difference in plant taxa, more investigations involving different plants are needed in order to unravel possible general rules. Moreover, an allopolyploid genome that possesses distinct properties from those of both a pure-line and a F1 hybrid has not been assessed for its possible differential response with regard to genetic and epigenetic stability to tissue culture.

In this study, we investigated tissue culture-induced genetic and epigenetic alterations in a set of rice (*Oryza sativa* L.) genotypes including two pure-lines (different subspecies, *japonica* and *indica*), a pair of reciprocal F1 hybrids parented by the two pure-lines, and a pair of reciprocal tetraploids resulted from the hybrids. There are dual advantages to use the rice system for this investigation, i.e., its unrivaled rich genomic information as a monocot model, and its status as a staple food crop for two-thirds of the human population [15,16]. We aimed to explore whether and to what extent these three distinct types of rice genotypes may respond differentially to the tissue culture condition with respect of genetic and epigenetic alterations, and their possible connectivity with perturbed expression of critical genes involved in DNA repair and DNA methylation.

## Results

### Genetic alteration in calli and regenerants of rice pure-lines, their reciprocal F1 hybrids, and tetraploids

**Figure 1:**
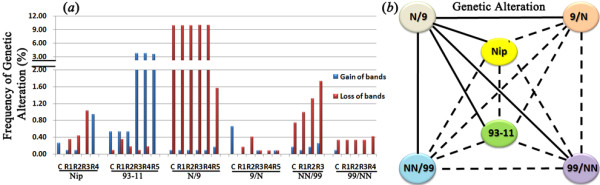
**Genetic alteration induced by tissue culture in rice.** (**a**) Frequencies of genetic alterations detected by amplified fragment length polymorphism (AFLP) in calli (C) and independent regenerated plants (regenerants, R_n_) in each of the six rice genotypes: two pure lines, Nipponbare (Nip) and 93–11, a pair of reciprocal F1 hybrids (N/9 and 9/N) parented by the two pure-lines, and a pair of reciprocal tetraploids (NN/99 and 99/NN) resulted from the F1 hybrids. Two types of alteration patterns, loss of parental bands and gain of novel bands are shown. (**b**) Diagrammatic illustration of the genotypic differences between the pairwise comparisons for ”Frequency of genetic alteration”. Solid and dashed lines indicate statistically significant (*p* < 0.05) and insignificant (*p* > 0.05) differences, respectively, based on one-way ANOVA test (*see*Methods).

The AFLP maker has been widely used to detect length changes of restricted DNA fragments subsequent to PCR amplifications [13]. It is the method of choice for fingerprinting genomic instability from a global perspective without entailing large-scale (re)sequencing. By using 18 pairs of selective primer combinations (Additional file 1), 1,165, 1,142, 1,221, 1,227, 1,216, and 1,215 reproducible bands (between two technical replicates, *see*Methods) were resolved for three kinds of samples (seed-plants, calli and regenerants) from each of the six genotypes. These six genotypes included two pure-lines (Nipponbare and 93–11, of two subspecies, *japonica* and *indica*, respectively), a pair of reciprocal F1 hybrids between the two lines (N/9 and 9/N), and a pair of reciprocal tetraploids resulting from the hybrids (NN/99 and 99/NN). Alteration in the AFLP banding patterns was detected in both calli and regenerants of all six genotypes compared with their respective seed-plants as controls. The alterations can be divided into two types, loss (disappearance of original bands of seed-plant control) and gain (*de novo* appearance of novel bands) (Figure 1a; Additional file 2). Due to the co-dominant nature of the AFLP marker, loss can be detected in the pure-lines and tetraploids only if both copies of the locus in question have been altered, but loss of one copy is detectable in the F1 hybrids if the locus was heterozygous. Thus, frequencies of the loss-type alteration would have been under-estimated in the pure-lines and the tetraploids relative to the hybrids. Indeed, in one of the pure-lines (93–11), the gain-type alteration is predominant, but this is not apparent in the other pure-line (Nipponbare) in which the alteration type is mainly dependent on the different regenerants (Figure 1a).

The most dramatic difference in genetic alteration frequencies are observed between the pair of reciprocal F1 hybrids, with calli and regenerants of N/9 (Nipponbare as maternal parent) showing more than 30-fold more alterations than those of 9/N (93–11 as maternal parent), and nearly all the alterations are of the loss-type (Figure 1a; Additional file 2). For the pair of reciprocal tetraploids, although calli and regenerants of NN/99 (whole-gnome doubling, or WGD of N/9) also showed higher frequencies of alteration than those of 99/NN (WGD of 9/N), the difference is much smaller in magnitude than that between the reciprocal hybrids, and the alterations are also predominantly of the loss-type (Figure 1a; Additional file 2). Across all six genotypes, it appears that the F1 hybrid N/9 showed the highest frequencies of genetic alteration (almost exclusively of the loss-type), followed by pure-line 93–11 (predominantly of the gain-type), while all the rest four genotypes showed much lower frequencies of alterations. However, it is notable that although the pair of tetraploids (NN/99 and 99/NN) showed similarly lower genetic alteration frequencies (relative to F1 hybrid 9/N) as the two pure-lines, they are of distinct types, i.e., predominantly band loss in tetraploids *vs.* band gain in pure-lines (Figure 1a).

To further test if the appeared genotypic differences in generating the genetic alterations as a result of tissue culture are statistically significant, we performed ANOVA and LSD-based multiple comparisons by including all regenerants for each of the six genotypes. We found that N/9 is indeed significantly different from all the rest five genotypes, while differences in all the rest pairwise comparisons of the five genotypes did not reach a statistically significant level (Figure 1b), verifying that N/9 is uniquely prone to generating genetic alterations under the tissue culture condition.

### Epigenetic alteration in calli and regenerants of rice pure-lines, their reciprocal F1 hybrids, and tetraploids

MSAP is a modified version of AFLP by substituting the *Mse*I enzyme with a pair of cytosine methylation-sensitive isoschizomers, *Hpa*II and *Msp*I. This pair of enzymes recognize the same tetranucleotide restriction site (5’-CCGG) but have differential sensitivity to methylation states of the two cytosines: *Hpa*II will not cut if either of the cytosines in the double-strand is methylated, whereas *Msp*I will not cut if the external cytosine is fully- or hemi- (single-strand) methylated [17]. Thus, for a given DNA sample, the internal cytosine methylation in double-strand, or external cytosine methylation in single-strand, at the assayed 5’-CCGG sites, can be unequivocally identified by the MSAP marker [13]. For clarity, we hereby refer to these two types of patterns as CG methylation and CHG methylation, respectively.

**Figure 2:**
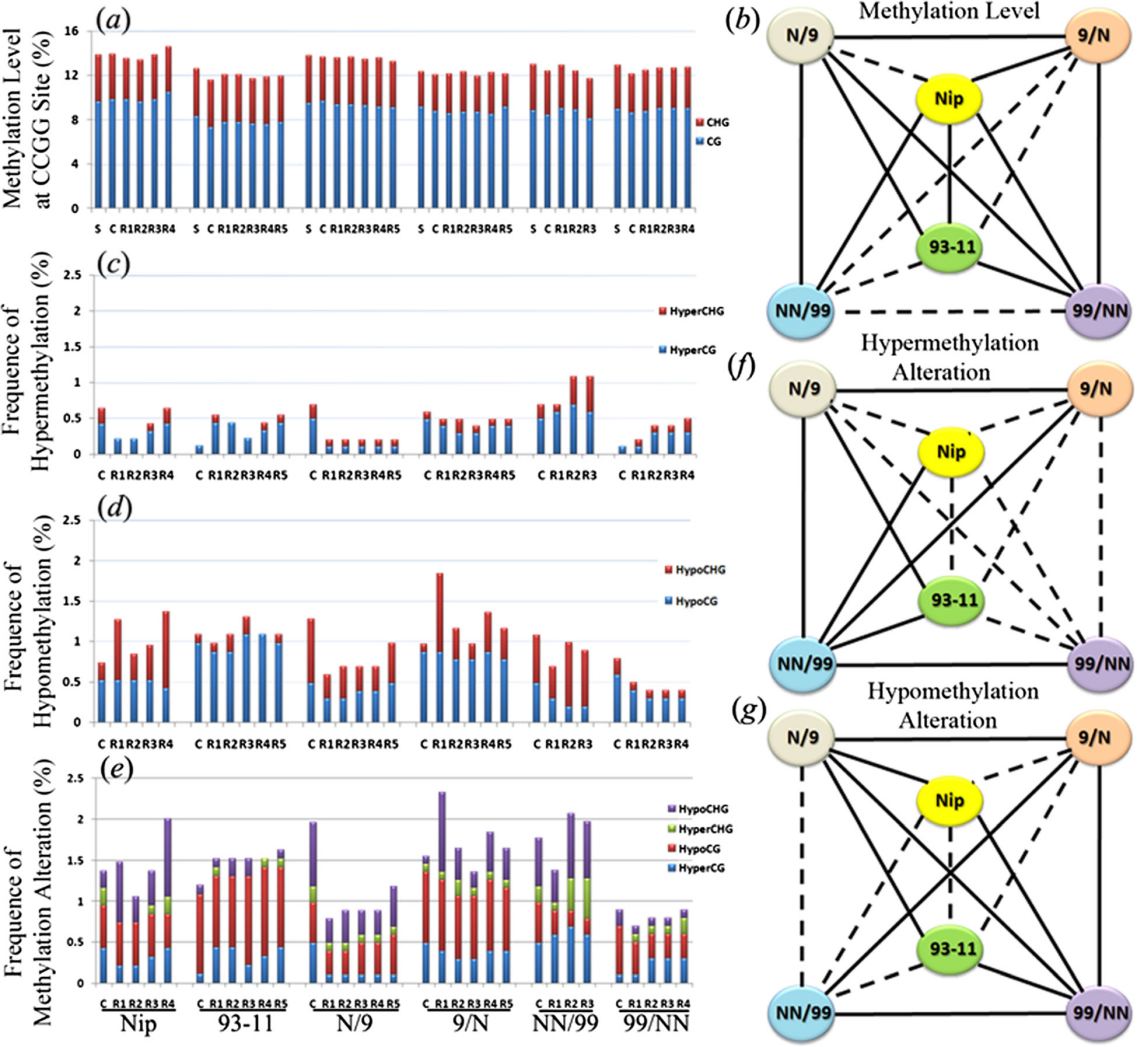
**Epigenetic alteration induced by tissue culture in rice.** (**a**) DNA methylation level of CG, CHG and total (adding up the two) at randomly sampled 5’-CCGG sites by methylation-sensitive amplification polymorphism (MSAP) in each of the six rice genotypes as in Figure 1. (**b**) Diagrammatic illustration of the genotypic differences between the pairwise comparisons for “Total methylation level” shown in (**a**). (**c**), (**d**) and (**e**) alteration in four major types methylation patterns, CG hyper and CHG hyper, CG hypo and CHG hypo, and collective alteration (adding up the four types) at randomly sampled 5’-CCGG sites by methylation-sensitive amplification polymorphism (MSAP) in each of the six rice genotypes as in Figure 1. (**f**) Diagrammatic illustration of the genotypic differences between the pairwise comparisons for “Total hypermethylation alteration” shown in (**c**). (**g**) Diagrammatic illustration of the genotypic differences between the pairwise comparisons for “Total hypomethylation alteration” shown in (**d**). In all cases, solid and dashed lines indicate statistically significant (*p* < 0.05) and insignificant (*p* > 0.05) differences, respectively, based on one-way ANOVA test (*see*Methods).

Using 16 pairs of selective *Eco*RI *+ Hpa*II/*Msp*I primer combinations (Additional file 1), 940,922,1,018,1,031,1,017 and 1,013 clear and reproducible MSAP bands (between two technical replications, *see*Methods) were scored for the three kinds of samples (seed-plants, calli and regenerants) of each of the six genotypes, as for AFLP. First, by tabulating the number of bands representing the two major methylation types, CG- and CHG-methylation, at the 5’-CCGG sites, we calculated the CG, CHG and total or collective (adding up the two) methylation levels in calli and regenerants (Figure 2a; Additional file 3). Clear fluctuations of both CG- and CHG-, and hence, total methylation levels at the assayed 5’-CCGG sites are clear across the six genotypes; in contrast, the methylation levels are largely constant among the three kinds of samples (seed-plants, calli and regenerants) within a given genotype (Figure 2a). Indeed, ANOVA and LSD-based statistical analysis indicates that 10 out of the 15 possible genotypic pairwise comparisons (Nip *vs.* 93–11, Nip *vs.* hybrid 9/N, Nip *vs.* tetraploid NN/99, Nip *vs.* tetraploid 99/NN, 93–11 *vs.* hybrid N/9, 93–11 *vs.* tetraploid 99/NN, hybrid N/9 *vs.* hybrid 9/N, hybrid N/9 *vs.* tetraploid NN/99, hybrid N/9 *vs.* tetraploid 99/NN, and hybrid 9/N *vs.* tetraploid 99/NN) showed significant difference in terms of the collective methylation levels (Figure 2b).

Next, we tabulated the alteration frequencies for further defined methylation patterns in calli and/or regenerants compared with their corresponding seed-plant controls of each genotype. Each of the CG and CHG methylation patterns can be further divided into two subtypes, i.e., hyper- and hypomethylation. Compared with their corresponding seed-plants, it is evident that calli and regenerants of all six genotypes showed both hyper- and hypomethylation alterations, which, in descending order, are CG hypo, CHG hypo, CG hyper, and CHG hyper (Figure 2c,d). Although difference clearly exists across the six genotypes, no distinct signatures are recognizable with regard to the three types of genotypes, i.e., pure-lines, F1 hybrids and tetraploids (Figure. 2c,d), which is in contrast with the genetic alterations (Figure 1a). Notably, taken all four subtypes of methylation patterns together, the alteration frequencies (Figure 2e) are markedly lower than those of the genetic alterations revealed by AFLP (Figure 1a). This reveals an important observation of this study, that is, in rice the major component of somaclonal variation is *genetic* rather than *epigenetic*. This contrasts with other studied plant species by the same methodologies, for examples, sorghum [13] and maize [14], in which the two types of alterations were found to occur at similar frequencies. Strikingly, calli and regenerants of hybrid N/9, which showed the highest frequencies of genetic alteration (Figure 1a) is among those showing the lowest frequencies of methylation alteration (Figure 2e), suggesting that tissue culture-induced genetic and epigenetic alterations in rice are uncoupled, and hence, likely controlled by distinct mechanisms, as also corroborated by the expression analysis of relevant genes, detailed in subsequent sections.

ANOVA and LSD-based statistical analysis was also conducted to test for significance of genotypic differences in the frequencies of tissue culture-induced hyper- and hypomethylation alterations. Results showed that (*i*) for frequencies of hypermethylation alteration, seven of the 15 pairwise comparisons (Nip *vs.* NN/99, 93–11 *vs.* N/9, 93–11 *vs.* NN/99, N/9 *vs.* 9/N, N/9 *vs.* NN/99, 9/N *vs.* NN/99, and NN/99 *vs.* 99/NN) showed statistically significant difference (Figure 2f) ; (*ii*) for frequencies of hypomethylation alteration, nine of the 15 pairwise comparisons (Nip *vs.* N/9, Nip *vs.* 99/NN, 93–11 *vs.* N/9, 93–11 *vs.* 99/NN, N/9 *vs.* 9/N, N/9 *vs.* 99/NN, 9/N *vs.* NN/99, 9/N *vs.* 99/NN, and NN/99 *vs.* 99/NN) showed statistically significant difference (Figure 2g).

### Chromosomal distribution and functional relevance of the genomic loci underlying tissue culture-induced genetic and epigenetic alterations

**Figure 3:**
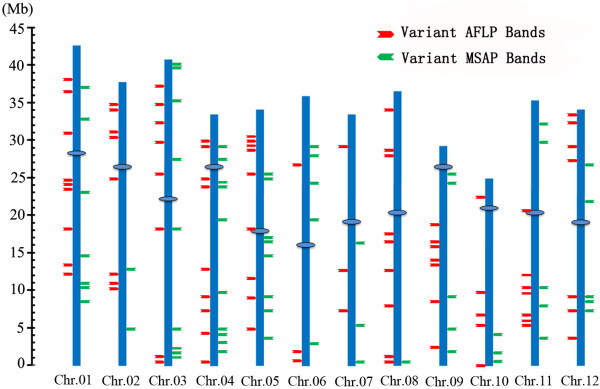
**Chromosomal locations of the sequenced variant AFLP bands (red arrows) and variant MSAP bands (green arrows) induced by tissue culture.** The mapping was based on a BlastN analysis against the rice whole genome sequence (http://rice.genomics.org.cn/rice/index2.jsp). Statistical test for nonrandom distribution of the two markers both within each of the 12 rice chromosomes was conducted by the Chi-squared test and the results were presented in Additional file 4.

To have a glimpse into the properties of the loci underwent genetic and methylation alterations as a result of tissue culture, we isolated, cloned and sequenced 86 and 67 variant bands from the AFLP and MSAP profiles, respectively. Based on the rice whole-genome reference sequence (http://rice.genomics.org.cn/rice/index2.jsp), 84 (97.7%) and 63 (94.0%) variant bands were mapped to unique loci across the rice chromosomes (Figure 3), indicating majority of these loci are single- or low-copy. To further test the random or nonrandom distribution of these variable loci among the rice chromosomes, the Chi-squared t-test (χ-test) against the null hypothesis of random distribution was performed by comparing the theoretically expected (E) and experimentally observed (O) values. The difference was insignificant between the expected and observed values (AFLP: *p* = 0.969 > 0.05, MSAP: *p* = 0.844 > 0.05) (Additional file 4), indicating that both genetic and epigenetic alterations induced by tissue culture are randomly distributed among the rice chromosomes.

To explore whether the variant AFLP and MSAP loci might bear functional relevance, we performed a BlastX analysis for the 86 variant AFLP bands and 67 variant MSAP bands, respectively, at the NCBI website (http://blast.ncbi.nlm.nih.gov/Blast.cgi). We found that two variant AFLP bands and one MSAP band showed significant homology to known-function genes, 44 AFLP bands and 23 MSAP bands were related to putative protein-coding genes, seven bands (three of AFLP and four of MSAP) showed homology to low-copy transposable elements (TEs), 36 AFLP bands and 40 MSAP bands showed no homology to the current rice database (Table 1; Additional files 5 and 6). The three known-function AFLP and MSAP bands are acetyltransferase NSI, mitogen activated protein kinase and ubiquitin-conjugating enzyme, respectively. This suggests that both the genetic and epigenetic alterations induced by tissue culture in rice are likely impacting biochemistry and physiology of the calli and regenerants, and hence, might be manifested as somaclonal variation at the phenotypic level.

**Table 1:** Functional classification of sequenced AFLP and MSAP variant bands in calli and/or regenerants

**Category (Based on BlastX)**	**Number and (%) of variant**	**Number and (%) of variant**
	**AFLP bands**	**MSAP bands**
Known-function gene	2 (2.3)	1 (1.5)
Putative protein-coding gene	44 (51.2)	23 (34.3)
Transposon and retrotransposon	4 (4.7)	3 (4.5)
No similarity	36 (41.9)	40 (59.7)
Total	86 (100)	67 (100)

### Altered transcript abundance by tissue culture of genes encoding for enzymes involved in DNA repair and DNA methylation

It has been suggested that a major cause for tissue culture-induced genetic and epigenetic alterations can be attributed to the breakdown of normal cellular controls for genetic and epigenetic fidelity [5]. It is known that three major repair pathways play essential roles in maintaining genetic fidelity under normal conditions, namely, somatic homologous recombination (SHR), mismatch repair (MMR) and cell-cycle checkpoint (checkpoint) [18-21]. Thus, it is conceivable that genetic alterations induced by tissue culture are probably related to perturbed- or mis-expression of the genes encoding for enzymes of these pathways under the condition. Similarly, the level and pattern of cytosine methylation are established and maintained in plants by interlaced actions of at least three categories of enzymes, i.e., cytosine methyltransferases, active demethylases and several Argonaute proteins (AGOs) related to biogenesis of a specific type of 24 nt small interference (si) RNAs [22-24]. Accumulating evidence also indicates that the at least the active demethylating process by the demethylases (DME) also involves nucleotide excision repairs [25,26], while it has been established that methylated and unmethylated cytosines have different propensities for single nucleotide transversion mutations via deamination [27]. Together, it is conceivable that tissue culture-induced alterations in DNA sequence and methylation may result from changed expression and/or activity of one or more of all the aforementioned enzymes under the condition. In addition, it also can be envisioned that some or all these cellular machineries would intrinsically interact, and produce the collective results of both genetic and epigenetic instabilities.

**Figure 4:**
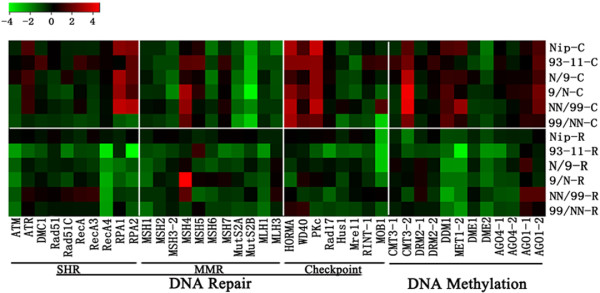
**Alteration in the relative steady-state transcript abundance for a set of genes involved in DNA repairing and DNA methylation in calli and regenerants relative to their corresponding seed-plants as controls for each of the six rice genotypes (detailed in Figure**1**) based on q-RT-PCR analysis.** A total of 41 genes were analyzed, which included those encoding for somatic homologous recombination proteins (SHR, 10 genes), mismatch repair proteins (MMR, 11 genes), checkpoint proteins (eight genes), DNA methyltransferases (six genes), 5-methylcytosine DNA glycosylases (two genes), and siRNA biogenesis-related proteins (four genes). Three rice house-keeping genes, a β-actin genes (Genbank accession no. X79378), a gene encoding for a protein synthesis elongation factor 1A, eEF-1a (Genbank accession no. AK061464) and a ubiquitin gene UBQ5 (Genbank accession no. AK061988), were used as internal controls for normalization. The steady-state transcript abundance for these genes are presented by fold-change (in log_2_) of original data (Additional file 8).

To assay possible perturbed expression by tissue culture of the aforementioned genes, we measured the steady-state transcript abundance for representative members in each of these four cellular pathways involved in DNA repair and DNA methylation in calli and regenerants relative to their corresponding seed-plant controls, for each of the six genotypes, by quantitative real-time RT-PCR (q-RT-PCR) analysis (for detailed information of genes and their primers, see Additional file 7). The analyzed genes include 10 of SHR, 11 of MMR, eight of checkpoint and 12 of DNA methylation. Fold changes (2^–ΔΔCt^) of the transcript abundance of these genes in calli and regenerants relative to their corresponding seed-plant controls were tabulated based on three replications (Additional file 8) and summarized (in log_2_) (Figure 4). The following observations can be generalized. (*i*) Compared with their corresponding seed-plant controls, both calli and regenerants showed significantly altered expression for most of the analyzed genes of each pathway (Figure 4; Additional file 8). (*ii*) While both up- and down-regulations are similarly prominent in calli, predominantly down-regulation was observed in regenerants (Figure 4; Additional file 8). (*iii*) Although the difference among pathways is not clear-cut, difference among individual genes is conspicuous, and which is largely concordant across the genotypes (Figure 4). (*iv*) Most strikingly, the difference in the expression of many of these genes between a pair of reciprocal F1 hybrids (N/9 *vs*. 9/N) and tetraploid (NN/99 *vs.* 99/NN) is also as great as that between the two pure-lines (Nipponbare *vs.* 93–11) in both calli and regenerants (Figure 4; Additional file 8), mirroring the results of genetic alterations (Figure 1). To explore whether this striking parent-of-origin difference in the altered expression of these genes is due to the effect of tissue culture or already pre-existing in seed-plants, we compared the expression of all these genes in the leaf-tissue of seed-plants from these six genotypes. We found that the parent-of-origin difference also exists among the seed-plants, but to a much lesser extent (Additional file 9). This suggests that expression of these genes of the four pathways entailed to maintain genetic and epigenetic stability are intrinsically sensitive to parent-of-origin effects even under normal conditions, but tissue culture-induced mis-expression substantially augments the preexisting parent-of-origin effect.

### Correlations between tissue culture-induced genetic/epigenetic alterations and altered transcript abundance of genes encoding for enzymes involved in DNA repair and DNA methylation

**Figure 5:**
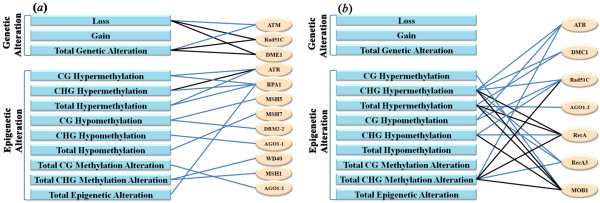
**Diagrammatic illustration of correlations between the tissue culture-induced various types of genetic/epigenetic alterations and altered expression of genes involved in DNA repairing and DNA methylation, in calli and regenerants, respectively.** A total of 41 genes were analyzed (detailed in Figure 4) and only those manifesting correlations with at lease one type of genetic or epigenetic alterations in calli or regenerants were shown. Pearson correlation analysis was performed and detailed in Additional file 10. The blue and black lines refer to significant correlations at levels of *P* < 0.05 and *P* < 0.01, respectively.

Although non-concordance was apparent between the tissue culture-induced genetic/epigenetic alterations (Figures 1 and 2) and the altered transcript abundance of genes encoding for enzymes involved in DNA repair and DNA methylation (Figure 4), it remains an intriguing possibility that some cryptic correlations might still exist between them, for example as specific types of genetic/epigenetic alterations and altered expression of specific genes or some sorts of collectivity thereof. To probe this, we conducted Pearson correlation analysis for all possible pairwise relationships. This analysis reveals that, first, there indeed exist significant correlations between the tissue culture-induced genetic/epigenetic alterations as specific types and altered expression of some of the analyzed genes encoding for enzymes involved in DNA repair and DNA methylation (Figure 5; Additional file 10). Second, more of the analyzed genes (12 out of 41) showed statistically significant correlations with the tissue culture-induced genetic/epigenetic alterations in calli than in regenerants (seven out of 41) (Figure 5; Additional file 10). Third, only three genes (ATR, Rad51C and AGO1-2) showed correlations with specific genetic and/or epigenetic alterations in both calli and regenerants, while correlations for the rest genes (nine in calli and four in regenerants) are confined to either calli or regenerants but not to to both (Figure 5; Additional file 10). Finally, none of the genetic alterations showed correlation with any of the gene expressions in regenerants, while most of the epigenetic alteration patterns showed even more correlations with altered expression of the genes in regenerants than in calli (Figure 5; Additional file 10). The last result makes sense because all the genetic alterations detected in the regenerants should have occurred at the callus stage while the occurrence of epigenetic alterations is not confined to the callus phase but also in the regeneration process, due to developmental dynamics of DNA methylation patterns in plants [13].

## Discussion

Although the molecular basis underlying the phenomenon of somaclonal variation remains to be fully understood, an array of recent investigations have provided substantive evidence that both genetic and epigenetic mechanisms are involved [8,10,11,28-30]. This is consistent with the earlier insights perceived by Phillips and colleagues that plant tissue culture-induced variation is a self-imposed mutagenesis as a consequence of disrupted normal cellular controls [5]. For example, a detailed investigation in *Arabidopsis* showed that expression of a large number of genes involved in chromatin regulation was preferentially altered (predominantly up-regulated) in cell suspension cultures relative to the seedling plants, and which was found to be concomitant with a shift in profiles of the two size-groups of siRNAs (21 nt *vs.* 24 nt) that play distinct regulatory roles [31]. It was suggested that the mis-regulation of chromatin genes and hence loss of epigenetic targeting (by siRNAs) under the tissue culture condition is largely responsible for the occurrence of genomic instability in the cell suspension cultures [31]. In parallel, genome re-sequencing analysis indicated that nucleotide changes, including single base substitutions (SNPs) and small insertion/deletions (indels) with distinctive molecular spectra from those of spontaneously accumulated natural mutations, have occurred extensively in regenerants of both *Arabidopsis*[15] and rice [7]. Interestingly, although reactivation of transposable elements (TEs) by tissue culture has been considered a general phenomenon associated with plant tissue culture [32-37], especially well-documented in rice [7,38-40], this was found totally absent in *Arabidopsis*[15], pointing to fundamental differences in the nature of molecular underpinnings of somaclonal variations in different plant taxa.

From an evolutionary point of view, merging of two divergent genomes (hybridization) and doubling of an existing genome, either of one genome (autopolyploidy) or a hybrid genome (allopolyploidy) represent revolutionary events often with cardinal biological effects, as they may lead to rapid organismal diversification and even stasipatric speciation [41,42]. A hallmark of nascent hybrid and polyploid genomes compared with that of a pure-line is their liability to change, which may confer enhanced evolvability compared to that of a pure-line [41,43]. Thus, the issue of whether and to what extent a nascent or established homoploid hybrid or polyploid genome would differ from that of a pure-line under various environmental conditions has drawn much attention in recent years [12,42]. This is because, apart from apparent evolutionary significance, the issue bears applied implications given that many important agricultural crops are hybrids or polyploids or their derivatives [41]. Given the unique attributes of plant tissue culture [1-3,5], it is of clear interest to explore the possible differences in response to this process between the distinct genomes of pure-lines, hybrids, and polyploids.

We recently found in sorghum that there exists a dramatic difference in frequencies of both genetic and epigenetic alterations induced by tissue culture between F1 hybrids and their parental pure lines, with the former being significantly more stable than the later [13]. In contrast, a set of maize inbred lines and their F1 hybrids did not show distinct differences in tissue culture-induced genetic and epigenetic alterations based on the same analytic tools [14], underscoring, again, differences in different plant taxa responding to tissue culture. The discrepancy also calls for more investigations using additional plant taxa for the purpose of unraveling possible generalities. Here, using six genotypes of rice which included two pure-line cultivars representing the *japonica* and *indica* subspecies of *Oryza sativa* L., a pair of reciprocal F1 hybrids parented by the two pure-lines, and a pair of reciprocal tetraploids resulted from the pair of F1 hybrids, we have further explored the issue, and extended the earlier study scope by including also polyploids. Several novel observations were made: (*i*) both genetic and DNA methylation alterations were detected in calli and regenerants from all six genotypes, but genetic alterations are more prevalent than epigenetic alterations; (*ii*) significant genotypic difference was observed in frequencies of both types of alterations, but only genetic alteration showed distinctive features among the three types of genomes, with one hybrid (N/9) showing an exceptionally high frequency of alteration; (*iii*) most surprisingly, difference in genetic alteration frequencies between the pair of reciprocal F1 hybrids is much greater than that between the two pure-line subspecies, and this strong parent-of-origin effect was significantly attenuated in the pair of reciprocal tetraploids; (*iv*) the steady-state transcript abundance of genes involved in DNA repair and DNA methylation was significantly altered in both calli and regenerants, and some of which were correlated with the genetic and/or epigenetic alterations. We caution that although our results are based on molecular marker analysis of relatively lager numbers of randomly sampled loci (*ca.* 1,000 for each of AFLP and MSAP), they are certainly pale compared with whole genome sequencing-based approaches. Nonetheless, our results have provided fresh testable entries for future more in-depth investigations on the molecular mechanisms of plant somaclonal variation by using systems biology approaches including whole genome re-sequencing, transcriptome profiling, methylome analysis.

## Conclusions

We have shown in this study that although molecular level somaclonal variation occurred in all three kinds of distinct rice genotypes, i.e., pure-lines, F1 hybrids and tetraploids, there exist fundamental differences among them in their propensities of generating both genetic and epigenetic alterations under the tissue culture condition. Genetic alteration is the major constituent of somaclonal variation in rice, and which is concomitant with epigenetic alteration in the form of cytosine DNA methylation patterns. In addition, parent-of-origin effect plays a major role in the genetic alteration frequencies of the pair of reciprocal hybrids, thus further implicating partitioning of epigenetic mechanisms in somaclonal variation, although which can be manifested as mainly genetic alterations. This intricate genetic and epigenetic interlacing is further corroborated by the strong association of perturbed expression of genes encoding for enzymes involved in DNA repair and DNA methylation with both genetic and epigenetic alterations.

## Methods

### Plant lines

The six rice lines used in this investigation included two rice pure-line cultivar, Nipponbare (*Oryza sativa* L. ssp. *japonica*) and 93–11 (*Oryza sativa* L. ssp. *indica*)*,* their reciprocal F1 hybrids (Nipponbare/93-11 and 93-11/Nipponbare, the first line being the maternal parent) and two tetraploids (NN/99 and 99/NN) resulted from the pair of F1 hybrids by colchicine-mediated whole-genome doubling. Individual plants of two pure-line cultivars were maintained by strict selfing in our laboratory for many generations before used for the F1 hybrids and tetraploids construction. Authenticities of constructed plants were validated by both cytology and molecular marker analysis before tissue culture.

### Tissue culture and regeneration

Calli were induced from germinating seeds of the six lines on Murashige-Skoog solid medium containing 2 mg/L 2,4-D. After incubation at 26 ± 1°C in darkness for about one month, calli were collected and transferred into NMB solid medium. After two months cultured at 26 ± 1°C in darkness, embryogenic calli were selected and subcultured on modified NMB medium and cultured at 26 ± 1°C under a 14h photoperiod for plant regeneration [44]. Regenerated shoots over 5 cm in height were transferred onto a rooting medium (growth-regulator free half strength MS medium containing 2% (w/v) sucrose and 0.68% (w/v) agar for root development and shoot strengthening. When grown to ~10 cm in height, the plantlets with healthy roots were removed from medium, rinsed in tap water and transplanted into a mixture of disinfected soil and grown under normal greenhouse conditions.

### DNA extraction, AFLP and MSAP analysis

Genomic DNA was isolated from expanded leaves by the high-salt CTAB method [45]. The standard amplified fragment length polymorphism (AFLP) protocol [46] was followed with modifications for silver staining [13]. The methylation-sensitive amplified fragment length polymorphism (MSAP) protocol was exactly as described [47]. For both markers, two technical replications (starting from independent DNA isolation) were performed and only clear and completely reproducible bands were scored.

### Recovery and sequencing of variant AFLP and MSAP bands

Bands showing various patterns of genetic or methylation alterations in calli and/or regenerated plants (regenerants) relative to the donor seed-plants were eluted from the silver-stained AFLP or MSAP gels and re-amplified with the appropriate selective primer combinations. The sizes of the PCR products were verified by agarose gel electrophoresis and then cloned into the pMD18-T vector (Takara Biotech. Inc., Dalian, China). The cloned variant bands were sequenced with vector primers by automated sequencing. The Advanced BlastN and BlastX programs at the NCBI website (http://www.ncbi.nlm.nih.gov/) were used for homology analyses. The cloned variant AFLP and MSAP bands were mapped *in silico* based on the rice whole genome sequence (http://rice.genomics.org.cn/rice/index2.jsp).The Chi-squared test was used to test for the random or nonrandom distribution of the variant bands across the 12 chromosomes of the rice genome (Additional file 4).

### Quantitative real-time-reverse transcriptase (RT) -PCR analysis

Total RNA was isolated using Trizol reagent (Invitrogen) according to the manufacturer's instructions and then treated with RNase-free DNase I (New England Biolabs) to remove any contaminating genomic DNA, and the reverse transcription (RT) reaction was performed using an RT system (Invitrogen, Carlsbad, USA) following the manufacturer’s protocol. Based on functional similarity, 41 genes were selected which included genes encoding for putative DNA methyltransferases (six), 5-methylcytosin DNA glycosylases (two), siRNA-related proteins (four), somatic homologous recombination proteins (SHR, 10), mismatch repair proteins (MMR, 11) and checkpoint proteins (eight). The house keeping gene β-actin, eEF-1a and UBQ5 were used as internal control. Primers used for q-RT-PCR were designed by the Primer 5 program (Premier Biosoft International, Palo Alto, USA). Forty-four pairs (including three for housekeeping genes used for normalization) of high quality primers with amplification efficiencies in the range of 92.5% to 104.9% were selected for study. The sequences, expected amplicon sizes and amplification efficiencies for all the primer pairs were presented in Additional file 7. The q-RT-PCR amplification was performed using an Applied Biosystems StepOnePlus™ Real-Time PCR System (Applied Biosystems, Foster City, USA) and the SYBR Premix Ex Taq (Takara) as a DNA-specific fluorescent dye. Conditions of q-RT-PCR were as reported [13]. Data were analyzed by using the software provided by ABI Company (Applied Biosystems, Foster City, USA) and calculated by the 2^–ΔΔCt^ method, according to previous studies [48,49].

### Statistics

The one-way ANOVA analysis was performed to statistically test for genotypic differences in the tissue culture-induced genetic and epigenetic variations, including ‘genetic alteration’, ‘methylation level difference’, ‘hypermethylation alteration’ and ‘hypomethylation alteration’, by analyzing the three or five independent regenerants from each of the six rice genotypes. Correlations were tested using the Pearson correlation analysis based on the data for alteration frequencies calculated based on the AFLP and MSAP variant frequencies and altered gene expression. Specifically, the ‘bivariate correlation, two-tailed, correlation coefficients, Pearson’s’ within the software SPSS v.14.0 (SPSS Inc., USA) was used. For testing statistic significance in the gene expression differences of the q-RT-PCR data, the SPSS v.14.0 software package (SPSS Inc., USA) was used by applying the one-way ANOVA test. Means were compared by the Least Significant Difference (LSD); when the p-value was less than 0.05, the difference was regarded as statistically significant.

## Competing interests

The authors declare no conflict of interest exists.

## Authors’ contributions

BL designed research; XRW, RW, XYL, YB, CDS and NZ performed research; XRW, XMY, CMX and YZD analyzed data; and XRW, YZD and BL wrote the paper. All authors read and approved the final manuscript.

## Supplementary Material

Additional file 1 Adaptors, pre-selective primers and selective amplification primers used in AFLP and MSAP analysis. Click here for file

Additional file 2 **An example of AFLP profiles showing the two types of genetic alterations in calli (C) and regenerants (R) in two pure lines (93–11 and Nip) , their reciprocal F1 hybrids (N/9 and 9/N) and tetraploids (99/NN and NN/99). Lanes S, C and R denote for seed plants, calli and regenerants, respectively.** The filled and empty arrows indicate loss and gain of bands, respectively, in calli and/or regenerants compared with their corresponding seed-plants for a given genotype. The primer combination is *Eco*RI + ATC/*Mse*I + CAG. Click here for file

Additional file 3 **An example of MSAP profiles showing different types of epigenetic c alterations in calli (C) and regenerants (R) in two pure lines (93–11 and Nip) , their reciprocal F1 hybrids (N/9 and 9/N) and tetraploids (99/NN and NN/99). Lanes S, C and R denote for seed plants, calli and regenerants, respectively.** The filled arrows indicate alterations in DNA methylation pattern in calli and/or regenerants compared with their corresponding seed-plants for a given genotype. The primer combination is *Eco*RI + AGG &*Hap*II/ *Msp*I + TCG. Click here for file

Additional file 4 Statistical test for the nonrandom distribution of the variant AFLP and MSAP bands across the 12 rice chromosomes. Click here for file

Additional file 5 Sequence analysis of variant AFLP bands isolated from calli and regenerated plants of the six rice genotypes. Click here for file

Additional file 6 Sequence analysis of variant MSAP bands isolated from calli and regenerated plants of the six rice genotypes. Click here for file

Additional file 7 Primers used for q-RT-PCR analysis. Click here for file

Additional file 8 **Alteration in the relative steady-state transcript abundance for a set of 41 genes involved in DNA repairing and DNA methylation in calli and regenerants relative to their corresponding seed-plants as controls for each of the six rice genotypes.** The relative quantitative expression profiles (2^–ΔΔCt^) of 41 rice genes were analyzed by comparing calli and regenerants of all six genotypes with their corresponding seed plants. The relative abundance of transcripts for each of the studied genes was calculated upon normalization against three rice housekeeping genes β-actin (accession no. X79378), eEF-1a (accession no. AK061464) and UBQ5 (accession no. AK061988). Click here for file

Additional file 9 **Alteration in the relative steady-state transcript abundance for a set of 41 genes involved in DNA repairing and DNA methylation in seed-plants of the six rice genotypes, based on q-RT-PCR analysis.** The genotypes include: two pure lines, Nipponbare (Nip) and 93–11, a pair of reciprocal F1 hybrids (N/9 and 9/N) parented by the two pure-lines, and a pair of reciprocal tetraploids (NN/99 and 99/NN) resulted from the F1 hybrids. For details of the analyzed genes see Additional file 8. Click here for file

Additional file 10 Correlation analysis between the various types of genetic and epigenetic alterations in calli and regenerants, respectively, based on Pearson correlation analysis. Click here for file
