# Supplementary figures and images for: Tissue culture-induced genetic and epigenetic alterations in rice pure-lines, F1 hybrids and polyploids
Source: BMC Plant Biol. 2013 May 5;13:77. doi: 10.1186/1471-2229-13-77 (PMC3648424; doi:10.1186/1471-2229-13-77)

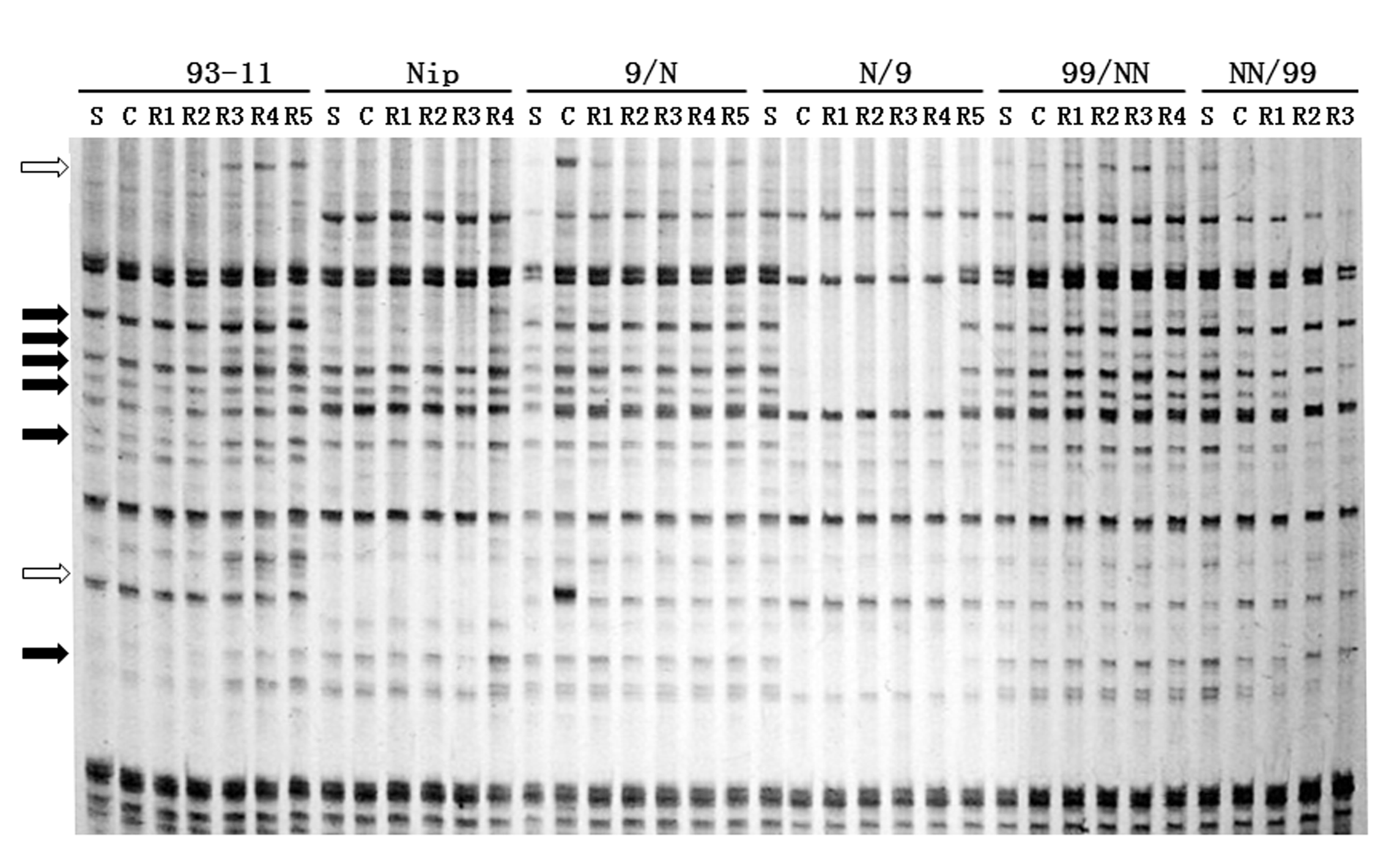

Supplement: Additional file 2 — An example of AFLP profiles showing the two types of genetic alterations in calli (C) and regenerants (R) in two pure lines (93–11 and Nip) , their reciprocal F1 hybrids (N/9 and 9/N) and tetraploids (99/NN and NN/99). Lanes S, C and R denote for seed plants, calli and regenerants, respectively. The filled and empty arrows indicate loss and gain of bands, respectively, in calli and/or regenerants compared with their corresponding seed-plants for a given genotype. The primer combination is EcoRI + ATC/MseI + CAG. [file 1471-2229-13-77-S2.tiff]

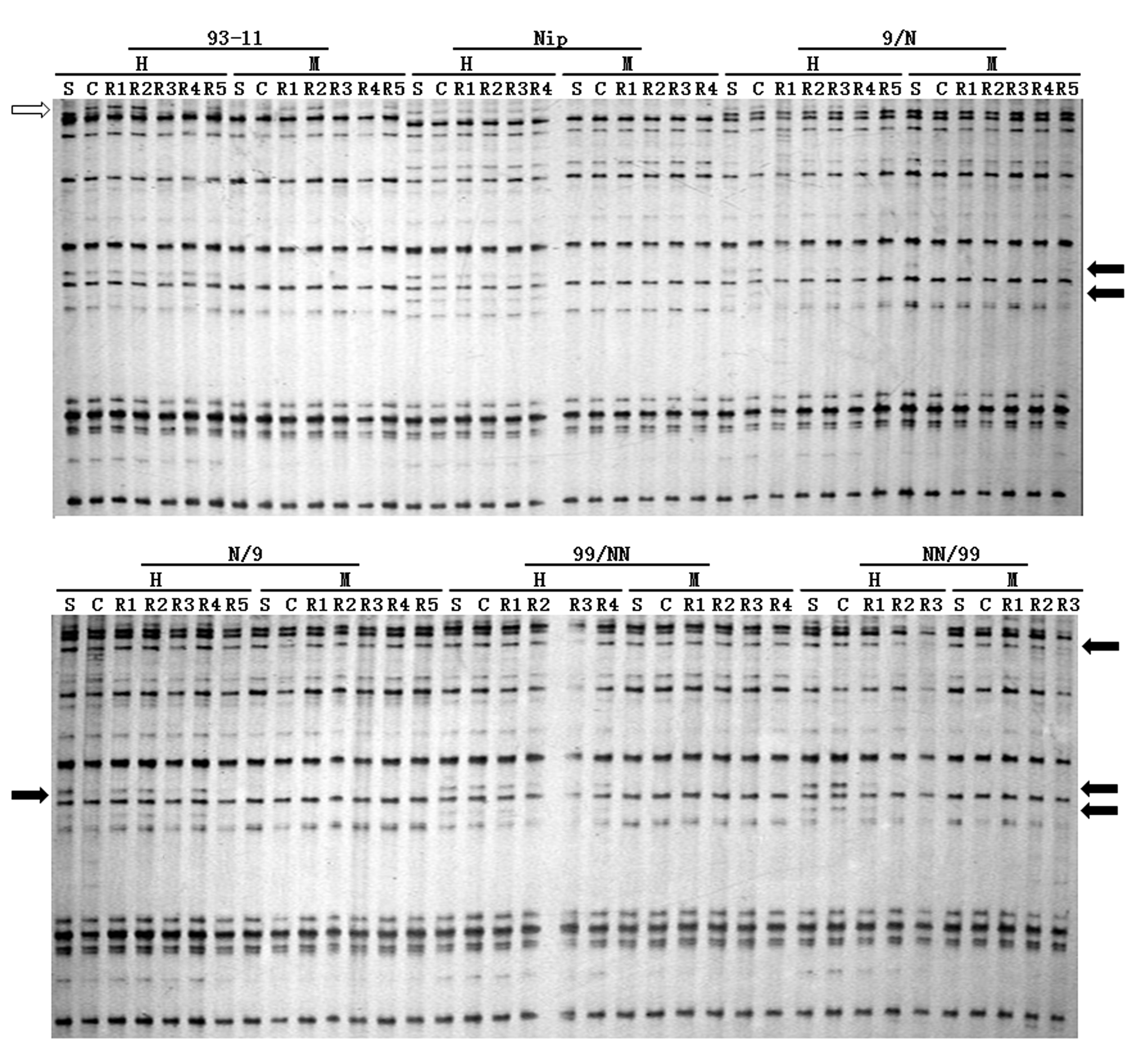

Supplement: Additional file 3 — An example of MSAP profiles showing different types of epigenetic c alterations in calli (C) and regenerants (R) in two pure lines (93–11 and Nip) , their reciprocal F1 hybrids (N/9 and 9/N) and tetraploids (99/NN and NN/99). Lanes S, C and R denote for seed plants, calli and regenerants, respectively. The filled arrows indicate alterations in DNA methylation pattern in calli and/or regenerants compared with their corresponding seed-plants for a given genotype. The primer combination is EcoRI + AGG &HapII/ MspI + TCG. [file 1471-2229-13-77-S3.tiff]

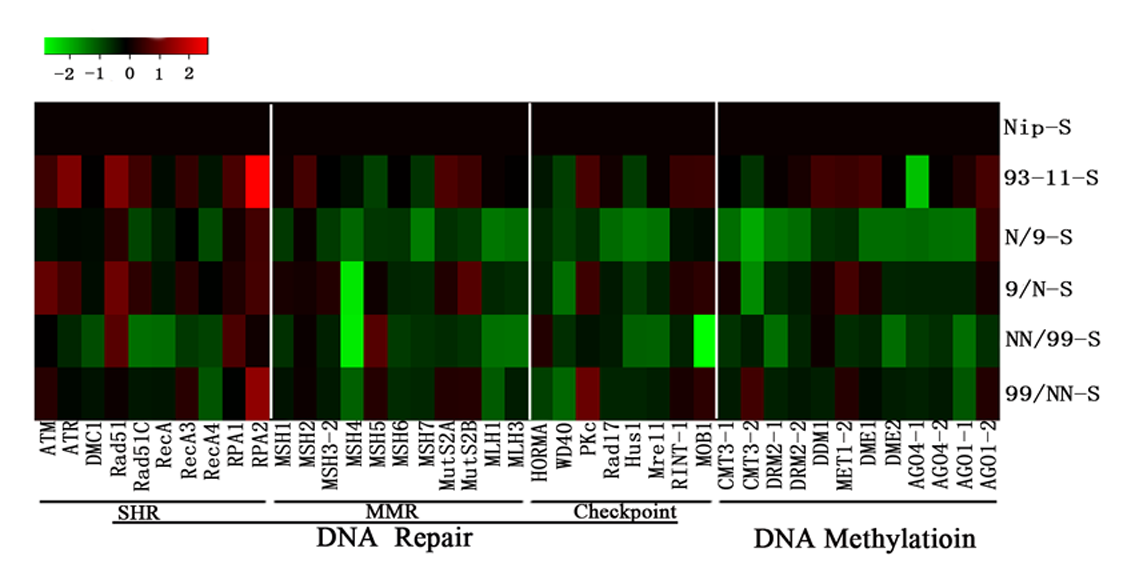

Supplement: Additional file 9 — Alteration in the relative steady-state transcript abundance for a set of 41 genes involved in DNA repairing and DNA methylation in seed-plants of the six rice genotypes, based on q-RT-PCR analysis. The genotypes include: two pure lines, Nipponbare (Nip) and 93–11, a pair of reciprocal F1 hybrids (N/9 and 9/N) parented by the two pure-lines, and a pair of reciprocal tetraploids (NN/99 and 99/NN) resulted from the F1 hybrids. For details of the analyzed genes see Additional file 8. [file 1471-2229-13-77-S9.tiff]
